# Hospital Notes

**Published:** 1895-05

**Authors:** 


### Hospital Notes.

The Samaritan Hospital for Women, Montreal, was thrown open for patients January 17, 1895. It is non-sectarian, and supported entirely by voluntary contributions, of which latter enough were handed in during the first month to carry on the work during a whole year. It is the only special hospital for diseases of women in Montreal, and will be molded on the pattern of the famous New York State Woman's Hospital in New York City. It is managed by a board of thirty of the principal ladies of the city, assisted by an advisory board of three laymen and three physicians. The staff consists of Sir James Grant, M. D., K. C. M. G., consulting physician ; Wm. H. Hingston, M. D., LL. D., consulting surgeon ; A. Lapthorn Smith, B. A., M. D., M. R. C. S., England, surgeon-in-chief ; H. Lionel Reddy, C. M., M. D., surgeon ; S. F. Wilson, C. M., M. D., assistant surgeon and registrar ; Dr. Sylvester, assistant surgeon, and Dr. Letellier de St. Just, assistant surgeon.

The Buffalo General Hospital has recently issued its thirty-sixth annual report, which is for the year 1894. It includes the report of the Ladies' Hospital Association, and the Training School for Nurses. It shows the prosperity and successful conduct of an institution that ought to be near the hearts of our citizens. It is to be hoped that the new hospital project will take substantial shape before another year passes.

